# Machine Learning-Based Detection of Pig Coughs and Their Association with Respiratory Diseases in Fattening Pigs

**DOI:** 10.3390/vetsci12090818

**Published:** 2025-08-26

**Authors:** Panuwat Yamsakul, Terdsak Yano, Kiettipoch Junchum, Wichittra Anukool, Nattinee Kittiwan

**Affiliations:** 1 School of Veterinary Medicine, Faculty of Veterinary Medicine, Chiang Mai University, Chiang Mai 50100, Thailand; terdsak.yano@cmu.ac.th (T.Y.); kiettipoch.junchum@gmail.com (K.J.); 2 Veterinary Research and Development Center (Upper Northern Region), Department of Livestock Development, Lampang 52190, Thailand; wiwanvet@gmail.com (W.A.); nattinee.k@dld.go.th (N.K.)

**Keywords:** machine learning, pig coughs, respiratory diseases, fattening pig

## Abstract

Respiratory infections are a major concern in pig farming as they negatively impact animal health and productivity. Coughing is a key symptom of respiratory disease and can be classified as productive or non-productive, but human assessment often leads to inconsistencies. This study aimed to use a machine learning model to classify pig coughs and investigate their association with respiratory infections. Cough sounds from 49 fattening pigs were recorded and analyzed using a Python-based machine learning system. The model’s accuracy in detecting coughs was 0.72, compared to 0.69 for farmers. For classification of non-productive coughs, the machine learning results showed strong agreement with infection status by *Mycoplasma hyopneumoniae*, with a Spearman’s correlation of 0.80 and a Cohen’s Kappa of 0.79. However, the association with Porcine Circovirus type 2 was weak, with correlation and Kappa values of 0.05 and 0.037, respectively. These findings indicate that machine learning can classify pig coughs more accurately than human evaluators and that non-productive coughs are strongly linked to Mycoplasma infection but not to PCV2. This suggests the potential use of machine learning for more reliable disease monitoring and early detection in swine production.

## Simple Summary

Respiratory problems in pigs are one of the important issues in pig farming, as they can affect both animal health and the overall productivity of the farm. Coughing is one of the noticeable symptoms and can be either productive or non-productive, but in practice, distinguishing between the two types can be difficult and often depends on the person’s experience. In this study, we aimed to use machine learning to help classify pig coughs and also find out if certain types of cough are linked with specific respiratory diseases. We recorded pig cough sounds and used a machine learning model to analyze them. We also compared its performance with the judgement of farmers and a pig specialist. The result showed that machine learning could classify coughs more accurately than people in most cases. Interestingly, we found that non-productive coughs are strongly related to one specific type of bacterial infection. This suggests that machine learning might be helpful as a tool for early detection of pig diseases in the future. We believe that this approach has potential to support swine health monitoring and improve disease management on pig farms.

## 1. Introduction

Respiratory problems are among the most common and critical health concerns in pig production systems. These conditions adversely impact key production indicators such as average daily gain (ADG), feed conversion rate (FCR), and mortality rate, ultimately leading to increased production costs [1] and reduced carcass quality. Many small-scale swine farmers are particularly vulnerable to these effects, with some even being forced to cease production altogether. The most important pathogens associated with respiratory diseases in pigs include Porcine Reproductive and Respiratory Syndrome virus (PRRSv), *Mycoplasma hyopneumoniae* (*Mh*), *Pasteurella* spp., Porcine Circovirus type 2 (PCV2), Swine Influenza virus, and *Streptococcus* spp. Among these, PRRSv and *Mh* have been identified as the primary causes of respiratory illnesses in pigs, both globally and in Thailand [2,3,4,5].

Porcine Reproductive and Respiratory Syndrome virus (PRRSv), an arterivirus, is classified into two main genotypes: PRRSV-1 and PRRSV-2 [6]. The virus primarily targets alveolar macrophages and respiratory epithelial cells, leading to productive cough and systemic manifestations such as reproductive failure in infected animals [5,7]. In Thailand, both genotypes are endemic, particularly in swine-dense regions [8]. In the present study, blood samples were selected for PRRSv detection due to their suitability in identifying both acute and chronic infections [9]. Reverse transcription polymerase chain reaction (RT-PCR), a method known for its high sensitivity and reliability, was employed for viral identification, even in asymptomatic or subclinical pigs [10,11]. Importantly, PRRSv is a key pathogen involved in the Porcine Respiratory Disease Complex (PRDC) and frequently co-infects with other respiratory pathogens such as Porcine Circovirus type 2 (PCV2) and *Mycoplasma hyopneumoniae* [12]. Surveillance data from Thailand indicate that PRRSv is present in over 70% of commercial pig herds. Similarly, PCV2 and Mh are reported with prevalence rates of approximately 60–80% and >50%, respectively. Although swine influenza virus (SIV) occurs less frequently (10–20%), its presence may exacerbate the complexity of respiratory disease diagnosis and management.

PCV2 is known to cause Postweaning Multisystemic Wasting Syndrome (PMWS), which leads to poor growth, emaciation, and chronic respiratory lesions [13,14]. Although its direct relationship to coughing is not well-defined, it plays a key role in respiratory disease complexity. In contrast, *Mh* is the causative agent of enzootic pneumonia, which is typically characterized by non-productive coughs. This pathogen adheres to tracheal cilia, disrupting mucociliary clearance and predisposing the lung to secondary infections [15]. Transmission occurs via aerosols or direct contact. Diagnostic samples often include lungs, trachea, or tonsils and are examined through PCR or culture. Importantly, different pathogens may produce distinct cough types: viral infections often cause productive coughs, while bacterial infections such as *Mh* typically result in non-productive coughs [16]. However, identifying cough type based on sound remains challenging due to the subjective nature of human perception [17]. In Thailand, respiratory diseases are highly prevalent in pig farms.

Because coughing is one of the earliest and most noticeable clinical signs of respiratory disease, the ability to detect and differentiate cough types accurately and early is crucial. Unfortunately, current diagnostic methods are reactive, invasive, and not always practical on commercial farms. This has driven interest in non-invasive, real-time surveillance tools. Syndromic surveillance, which emphasizes early recognition of abnormal signs before full outbreaks occur, provides a promising framework for such efforts. Recent technological developments in artificial intelligence (AI) have opened new possibilities for improving disease detection in swine production. AI has been used for activity monitoring, temperature analysis, weight estimation [18], and even analyzing pig vocalizations to assess stress or pain [19]. Importantly, machine learning algorithms have also been applied to classify pig coughing and background noise using spectrogram-based sound analysis [20].

This study builds upon these developments by investigating the use of machine learning to classify pig coughs into productive and non-productive types and to assess their association with specific respiratory pathogens. The findings could support early disease detection and more precise health monitoring in pig farms, contributing to improved welfare and productivity.

## 2. Materials and Methods

### 2.1. Animals and Sample Size for Coughing Pigs

Fattening pigs aged between 3 and 5 months, crossbred from Large White × Landrace and Duroc breeds, were selected from small-holder farms affiliated with the Chiang Mai–Lamphun Pig Farmers Cooperative Limited. These farms were known to have a documented history of coughing symptoms among their fattening pig populations. The required sample size of pigs exhibiting coughing symptoms was calculated using G*Power software (version 3.1). A one-tailed hypothesis was applied with a significance level (α) of 0.05 and a statistical power (1 − β) of 0.95. Based on these parameters, the minimum required sample size was determined to be 34 pigs. A total of 49 audio recordings were collected, including 25 productive coughs, 23 non-productive coughs, and one non-cough sound. All the participating farms provided consent for data collection, and the experimental protocol was reviewed and approved by the Animal Care and Use Committee of the Faculty of Veterinary Medicine, Chiang Mai University, Thailand (Approval No. S15-2567). To enhance the practical value of this study in the context of precision livestock farming, several aspects of real-world implementation were considered. These included the identification of optimal sensor placements within pig pens, the use of directional microphones to improve sound quality, the application of noise-filtering techniques to ensure signal robustness in high-noise environments, and a preliminary discussion of the cost–benefit trade-offs between adopting AI-based cough monitoring and conventional veterinary surveillance. These considerations further strengthen the applied relevance and feasibility of deploying the proposed system in actual farming conditions. This study employed a cross-sectional design to investigate the association between pig cough characteristics (productive vs. non-productive) and respiratory pathogens (*Mh*, PCV2, and PRRSv) in fattening pigs.

### 2.2. Recording of Pig Cough Sounds

The observation of coughing symptoms in fattening pigs was carried out at the participating farms. The pigs were stimulated to move around inside the pen for approximately 3 to 5 min, and then the coughing behavior was carefully observed by the farm owner at each farm. When a fattening pig was identified as coughing, it was marked with color spray as an indicator for further observation. After that, the pig farmer recorded the cough sound of the identified pig. Each sound file was labeled with a code that matched the specific pig that showed coughing symptoms. Before being processed using AI to convert the sound into a spectrogram image, each sound file was reviewed by an expert to confirm whether it was truly a pig cough or not. Furthermore, the sounds confirmed as pig coughs were then assessed by both the farmer and the expert to determine whether the cough was a productive cough or non-productive cough. The recorded and verified sounds were later analyzed in a studio room, where the files were converted and used for further classification and analysis of sound types.

### 2.3. Conversion of Sound Data into Images

**Figure 1:**
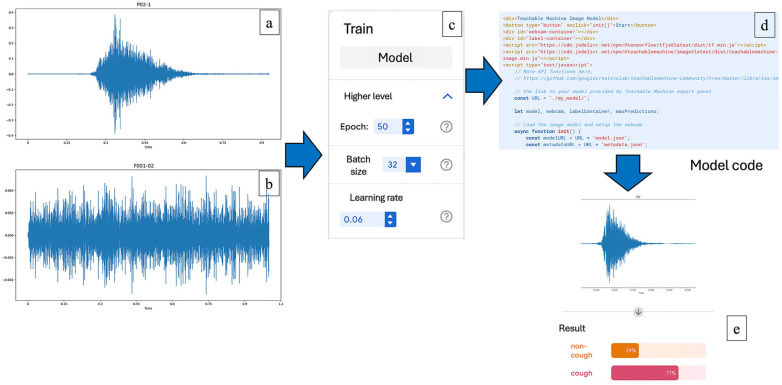
Training the AI system to analyze pig cough sounds: (**a**) a wave plot representing a reference pig cough sound; (**b**) a wave plot of a non-cough sound used as a comparison; (**c**) configuration parameters used in the AI training process via Google Teachable Machine, including 50 epochs, batch size of 32, and a learning rate of 0.06; (**d**) example of the model code used to deploy the trained AI model for classification; (**e**) output result from the AI analysis indicating classification confidence between “cough” and “non-cough” categories.

Each audio clip was independently evaluated by both a pig farmer and a swine practitioner. Only the files on which both parties reached consensus were included and labeled accordingly. For audio preprocessing, the Librosa library in Python (version 3.11.0) was employed to convert the sound files into Mel spectrograms with 128 mel-frequency bins. Each clip was standardized to a duration of 2.0 s with a sampling rate of 44.1 kHz. To improve signal quality, background noise was reduced using spectral gating. In addition, a band-pass filter between 500 Hz and 6000 Hz—representing the frequency range most commonly associated with pig coughing—was applied to further suppress irrelevant sounds. Stratified random sampling was used to divide the dataset into 80% for training and 20% for testing, ensuring that class distributions (productive cough, non-productive cough, and non-cough sounds) remained balanced across both sets. Finally, the sound files were converted into wave plot images using the Librosa library, with specific commands applied to ensure consistent formatting across samples. def plot_waves(y, sr):

file_path = ‘sound_name.wav’
y, sr = librosa.load(file_path)
fig = plt.figure(figsize = (60,25))
librosa.display.waveshow(np.array(y), sr = sr)
step = 0.2
duration = librosa.get_duration(y = y, sr = sr)
xticks = math.ceil(duration/step)
xticks = [x * step for x in range(xticks + 1)]
plt.suptitle(“Figure 1: Waveplot”, x = 0.5, y = 0.915, fontsize = 16)
plt.xlim(left = 0, right = 4)
plt.show()
plot_waves(y, sr)

#### Training the AI System

In this process, the AI system was instructed to classify the spectrogram images into two categories: cough and non-cough. Once the images identified as cough were separated, the AI was further trained to distinguish between productive and non-productive cough images. These classifications were used to analyze correlations in subsequent steps. The wave plot images obtained from the sound data were used to train the AI system. The training process was conducted using Google Teachable Machine. The model was trained for 50 epochs, with a batch size of 32 and a learning rate set at 0.06, which was identified as the optimal parameter configuration based on preliminary analysis.

**Figure 2:**
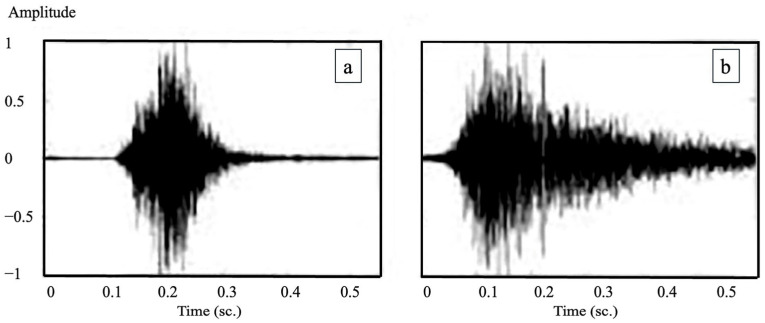
The wave plot comparison of cough sounds: (**a**) non-productive cough: monophasic amplitude pattern with reduced peak amplitude (0.5 to −0.5 arbitrary units); (**b**) productive cough: polyphasic oscillations with higher amplitude variability (adaptation from [1]).

In the next step, the system was trained to distinguish between non-productive and productive coughs (Figure 2). The method used was the same as the one for separating cough from non-cough sounds. However, the reference sounds used for the training were changed to clearly identified samples of either non-productive or productive coughs, which had been reviewed and agreed upon by both the farmers and the veterinary experts. These sounds were then converted into spectrogram images and used to teach the AI to recognize the differences between the two types. This allowed for more accurate classification of pig coughs and helped support further analysis of pig health conditions on the farms.

### 2.4. Collection of Blood and Tonsil Swab Samples

The pigs that showed coughing symptoms were restrained using a snare, which was placed between the upper and lower jaw teeth. Then, a mouth gag was inserted into the pig’s mouth to keep it open. After that, a sterilized cotton swab was used to rub the surface of the tonsil gland, and the swab was placed into BHL medium broth. The sample was kept at a temperature of 4–8 °C before being sent for laboratory analysis to detect *Mh*. For blood sample collection, 2 mL of blood was drawn from the pig’s jugular vein using a sterile EDTA tube. The blood samples were also kept at 4–8 °C and then sent to the laboratory for further diagnosis of PRRS virus and PCV2 virus.

### 2.5. Real-Time RT-PCR Method for Diagnosis of PRRS Virus

The detection of PRRS virus (PRRSv) was performed using the real-time reverse transcription polymerase chain reaction (real-time RT-PCR) technique. The process began with the extraction of viral RNA, followed by amplification of the target RNA using PCR, which allows the monitoring of RNA amplification in real time. RNA was extracted from the samples using a commercial RNA extraction kit. The extracted RNA solution was kept on ice during the entire testing process to prevent RNA degradation. The master mix for the one-step real-time RT-PCR reaction was prepared, including three control samples: positive control for type 1, positive control for type 2, and a negative control. The reaction was conducted using the LightCycler^®^ multiplex RNA virus master (Roche GmbH, Mannheim, Germany). The 20 µL one-step real-time RT-PCR reaction included the following components: (1) 4.0 µL of 5 × RT-qPCR reaction mix, (2) 0.1 µL of 200 × RT enzyme solution, (3) primers at a final concentration of 500–600 nM, and (4) probes at a final concentration of 250–300 nM.

The amplification of the target RNA was monitored in real time by detecting the increase in specific fluorescence signals. For PRRS virus type 1, fluorescence was detected in the Cy5 channel, while for type 2, the HEX channel was used (Table 1). The results were reported as cycle threshold (Ct or Cq), which indicates the cycle number at which a significant increase in fluorescence was observed. A typical amplification curve appeared in the form of an S-shaped (sigmoid or exponential) graph.

### 2.6. Quantitative PCR (qPCR) for Detection of Porcine Circovirus Type 2 (PCV2)

Detection of Porcine Circovirus type 2 (PCV2) DNA was performed using a quantitative PCR (qPCR) protocol adapted from Franzo et al. (2018) [22] and Yuan et al. (2014) [23] (Table 2). To begin with, DNA was extracted from tonsil swab samples preserved in broth medium using a commercial DNA extraction kit according to the manufacturer’s protocol. The extracted DNA was kept on ice during preparation to prevent degradation. Then, the qPCR reaction was prepared in a total volume of 20 µL, comprising the following components: 10.0 µL of 2 × qPCR reaction mix, 400 nM of each forward and reverse primer, and 200 nM of probe specific for PCV2. A total of 1.0 µL of DNA template or control (positive or negative), with nuclease-free water added to adjust to the final volume of master mix (12.5 µL per tube), was aliquoted into PCR tubes, followed by the addition of 1.0 µL of extracted DNA. Positive and negative controls were included in each run. The negative control consisted of nuclease-free water. Thermal Cycling Conditions: the qPCR was conducted using a real-time thermal cycler under the following conditions. Initial denaturation: 95 °C for 10 min (1 cycle); amplification: 95 °C for 15 s (45 cycles); cooling: 25 °C for 45 s (1 cycle). Then, fluorescence signals were monitored at each cycle to detect the specific amplification of the PCV2 target sequence. The results were reported based on the cycle threshold (Ct) values corresponding to exponential amplification curves.

### 2.7. Bacterial Culture and qPCR-Based Confirmation of Mycoplasma hyopneumoniae

The presence of *Mh* was initially assessed by culturing samples in BBL™ Mycoplasma broth (BHL medium) at 37 °C. The cultures were monitored for color change, which indicates metabolic activity. In the absence of visible color change, blind passages were performed every 5–7 days for a total of 21 days to enhance bacterial recovery [8]. DNA was extracted from the cultured broth using a commercial DNA extraction kit according to the manufacturer’s instructions. Quantitative PCR (qPCR) targeted the Mhp183 gene, which is specific to *Mh*, to confirm the presence of the pathogen (Table 3).

The qPCR amplification was performed under the following thermal cycling conditions: DNA polymerase activation at 95 °C for 15 min to activate the enzyme, 40 cycles of denaturation at 95 °C for 15 s, combined annealing and extension at 60 °C for 60 s.

### 2.8. Statistical Analysis of the Relationship Between Coughing Symptoms and Pathogen Detection

Three types of evaluators including pig farmers, an artificial intelligence (AI) system, and the swine practitioner expert were asked to interpret the wave plots in order to determine whether the sound represented a cough and, if so, to classify the type of cough. The results of the analysis were used to calculate the sensitivity and specificity of cough detection, as well as detection of non-productive coughs. These evaluations were performed for pig farmers vs. the AI system and the swine practitioner expert vs. the AI system. The accuracy of cough identification and cough type classification was then compared using Bayesian probability theory. Also, the classification results for non-productive coughs were statistically analyzed in relation to the PCR test results using Spearman’s correlation coefficient and the Kappa coefficient, implemented in the R statistical software (v 4.4.1). A *p*-value of less than 0.05 was considered statistically significant.

## 3. Results

### 3.1. Detection Results for Mycoplasma hyopneumoniae (Mh), Porcine Circovirus Type 2 (PCV2), and Porcine Reproductive and Respiratory Syndrome Virus (PRRSv)

Out of 49 tonsil swab samples, 10 samples tested positive for *Mh*. For Porcine Circovirus type 2 (PCV2), 29 out of 49 blood samples showed positive results. In contrast, no positive results were found for PRRSv in any of the tested samples.

### 3.2. Probability Analysis of General Cough Detection

The sensitivity and specificity of the pig farmers in identifying coughing sounds, as compared with the swine practitioner expert, were 1.00 and 0.056, respectively. For the AI-based cough detection system, the sensitivity and specificity were 1.00 and 0.50, respectively, when benchmarked against swine experts. Bayesian probability analysis was used to determine the likelihood that each system correctly identified cough events. The posterior probabilities for accurate cough detection by pig farmers and by the AI system were 0.31 and 0.46, respectively. These results suggest that although both observers and AI systems are capable of recognizing cough events (high sensitivity), the AI system demonstrates greater ability in avoiding false positives (higher specificity), thereby providing a more balanced and reliable diagnostic outcome.

### 3.3. Probability Analysis of Non-Productive Cough Detection

In the identification of non-productive coughs (i.e., dry coughs), the sensitivity and specificity of the pig farmers, compared to swine experts, were 0.80 and 0.85, respectively. The AI system showed slightly higher sensitivity (0.90) and equal specificity (0.85). Bayesian analysis of posterior probabilities revealed that the likelihood of correctly detecting a non-productive cough was 0.69 for the pig farmers and 0.72 for the AI system. These findings indicate that both human and AI systems have strong diagnostic performance in detecting non-productive coughs, with the AI system showing slightly better overall probability due to higher sensitivity.

### 3.4. Correlation Between Positive (PCV2 and Mh) and Coughing or Non-Productive Cough

The dataset was split into 80% for training and 20% for testing to validate the machine learning model. The model performance was evaluated using accuracy (0.72), precision (0.85), recall (0.90), F1-score (0.87), and ROC-AUC (0.89) metrics. To evaluate pathogen–cough relationships, Spearman’s correlation and Cohen’s kappa coefficients were calculated (Table 4).

The machine learning model demonstrated robust performance, having 0.89 ROC-AUC, significantly outperforming farmer assessments. While PCV2 showed negligible correlation with non-productive coughs (κ = 0.037), *Mh* exhibited strong agreement (κ = 0.79), reinforcing its pathognomonic relationship with dry coughs.

## 4. Discussion

Respiratory diseases in swine production remain one of the major concerns due to their significant impact on both animal welfare and farm productivity. Among the earliest clinical indicators, coughing is commonly observed; however, the identification and interpretation of cough characteristics are often subjective when solely dependent on human observation. In the present study, we investigated the feasibility of utilizing artificial intelligence (AI) to detect and classify pig coughs and examined their association with two key respiratory pathogens: *Mycoplasma hyopneumoniae* (*Mh*) and Porcine Circovirus type 2 (PCV2). Dry, non-productive coughing is a common clinical sign of *Mh* infection, particularly observable when pigs are physically stimulated to move, as has been reported in field observations [24]. Recent advances in digital sound analysis have enabled more objective cough characterization. In particular, spectrogram and wave plot analysis can reliably differentiate infectious cough sounds from ambient farm noises. Berckmans et al. (2008) [25] demonstrated that coughs from infected pigs exhibit distinctive wave plot signatures—characterized by increased duration and altered frequency content—when compared to normal coughs or environmental noises. These distinctions are especially evident when visualized using both time-domain and frequency-domain analyses. In our study, the classification of cough types into “productive” and “non-productive” was carried out by both experienced pig farmers and a swine health expert. To assess the reliability of these human-based annotations, inter-rater agreement was evaluated using Cohen’s kappa coefficient. The resulting kappa value suggested moderate agreement, indicating that although some degree of subjectivity remained, the classification approach was reasonably consistent.

Although PRRSv (Porcine Reproductive and Respiratory Syndrome virus) was included as one of the target pathogens, no PRRSv-positive cases were detected in the sampled population. This may be attributed to the relatively low prevalence of PRRSv in the selected geographic region during the study period. Additionally, the majority of sampled pigs were fattening pigs in the grower-finisher phase, which are typically more robust and exhibit fewer clinical signs compared to younger or immunologically naïve animals. The absence of PRRSv detection in this study may also reflect effective disease control measures implemented by the participating farms. Consequently, the current data were insufficient to evaluate the association between PRRSv infection and cough characteristics. Future studies involving larger and more diverse pig populations—including weaning pigs and herds with known PRRSv circulation—are recommended to further investigate this association.

The results demonstrated that the AI system outperformed pig farmers in identifying general coughing sounds (0.46 vs. 0.31) and was particularly more effective in detecting non-productive (dry) coughs (0.72 vs. 0.69). Although the difference appears modest, the AI’s consistent and objective ability to classify cough types offers a clear advantage, especially in large-scale or continuous monitoring contexts. This finding aligns with prior research showing that AI-based acoustic monitoring systems can significantly enhance real-time animal health surveillance. For example, Zhao et al. (2020) [26] developed a DNN–HMM acoustic model that effectively distinguished pig coughs, non-coughs, and silence segments with an improved Word Error Rate (WER), a standard metric for evaluating speech recognition performance [27]. Similarly, a systematic review found that microphone-based cough detection systems can achieve accuracies between 73% and 93% in detecting respiratory distress in pigs, demonstrating their potential for welfare and health monitoring [28].

When analyzing the relationship between cough type and pathogen detection, our findings indicated a moderate association between general coughing and combined detection of respiratory pathogens (PCV2 and *Mh*), with a Spearman’s rho of 0.40 and Cohen’s kappa of 0.36 (95% CI: 0.10–0.62). In contrast, the correlation between PCV2 and non-productive cough was weak (rho = 0.05; kappa = 0.037, 95% CI: –0.20–0.27), suggesting that PCV2 infection alone is not a reliable predictor of this clinical sign. These results align with previous reports demonstrating that PCV2 often plays a subclinical role or acts synergistically rather than as a primary respiratory pathogen. Notably, co-infection of PCV2 and *Mh* has been experimentally shown to exacerbate lung lesions in PRDC models [29,30]. Conversely, some findings report minimal lung pathology in PCV2-only infections. Such variability underscores the complex interplay between PCV2 and other respiratory pathogens, reinforcing the need for longitudinal field studies to better delineate these relationships [31].

Although PRRSv (Porcine Reproductive and Respiratory Syndrome virus) was included as a target pathogen in this study, no PRRSv-positive cases were detected among the sampled pigs. This may reflect the low prevalence of PRRSv in the study area during the sampling period or may be attributed to the fact that the sampled population consisted primarily of clinically stable fattening pigs. Importantly, the cough sounds observed in this study—particularly non-productive coughs—were more strongly associated with *Mh* infection than with PRRSv. This observation is consistent with previous clinical reports indicating that PRRSv often induces more systemic symptoms, including fever, anorexia, and reproductive failure, whereas dry, non-productive coughing is a characteristic sign of *Mh* infection. Therefore, the absence of PRRSv in the dataset is unlikely to have substantially affected the performance or clinical relevance of the AI-based cough classification system used in this study.

In contrast, a strong correlation was observed between *Mh* infection and non-productive coughs (Spearman’s rho = 0.80; kappa = 0.79, CI: 0.56–1.00). This finding is consistent with the pathophysiology of *Mh*, which typically causes dry, persistent coughing due to the organism’s ability to adhere to and damage ciliated epithelial cells in the respiratory tract [32,33,34]. In experimental co-infection studies, *Mh* has been shown to induce chronic coughing when combined with other pathogens such as PCV2, reinforcing its role in respiratory disease complexes [30]. Moreover, the high agreement between AI-detected dry coughs and laboratory-confirmed *Mh* infection supports the potential of AI systems as effective screening tools. Recent studies have demonstrated that deep learning models can detect pig coughs with high accuracy, sensitivity, and specificity under commercial farm conditions [35]. AI-based cough monitoring also enables earlier detection of *Mh* outbreaks, which is crucial for timely intervention and reducing antibiotic use [36]. The high kappa value observed in this study further reinforces the utility of AI as a proxy for clinical diagnosis, particularly in field settings where routine diagnostic testing is not always feasible.

These findings emphasize the usefulness of AI in pig farms, particularly for continuous and objective monitoring of respiratory symptoms. The ability to differentiate cough types and correlate them with specific pathogens like *Mh* provides a promising avenue for non-invasive early warning systems. However, some limitations should be noted. The relatively small sample size (*n* = 49) may have influenced the statistical power, especially regarding the PCV2 group. Additionally, environmental noise and the presence of multiple pigs in the same enclosure could affect acoustic detection accuracy.

## 5. Conclusions

In conclusion, this study highlights the potential of artificial intelligence systems in improving the detection and classification of coughs in pigs, with a particular strength in identifying non-productive coughs associated with *Mycoplasma hyopneumoniae*. Future research should focus on expanding sample sizes, incorporating real-time AI models in farm environments, and integrating multimodal data such as temperature, behavior, and video imaging to enhance diagnostic precision. Additionally, longitudinal studies can assess the predictive value of AI-detected coughs over time and determine whether such systems can be integrated into automated decision-support tools for veterinarians and farm managers.

## Figures and Tables

**Table 1:** Primer sequences and their specificity used for real-time RT-PCR detection of PRRSv (adapted from [21]).

Primer/Probe	Nucleotide Sequence (5′–3′)	Target (Specificity)
PRRS-Eu/F	GCA CCA CCT CAC CCR RAC	PRRSV Type 1 (ORF7)
PRRS-Eu/R	CAG TTC CTG CRC CYT GAT	PRRSV Type 1 (ORF7)
PRRS-Eu/Pr	Cy5-CCT CTG YYT GCA ATC GAT CCA GAC-BHQ1	PRRSV Type 1 (ORF7)
PRRS-Us/F	ATR ATG RGC TGG CAT TCC	PRRSV Type 2 (ORF7)
PRRS-Us/R	ACA CGG TCG CCC TAA TTG	PRRSV Type 2 (ORF7)
PRRS-US/Pr	HEX-TGT GGT GAA TGG CAC TGA TTG ACA-BHQ1	PRRSV Type 2 (ORF7)

Remake: ORF7 = Open Reading Frame 7.

**Table 2:** Primer and probe nucleotide sequences used for quantitative PCR (qPCR) detection of PCV2 and PCV3 [22,23].

Primer/Probe	Nucleotide Sequence (5′–3′)	Target (Specificity)
PCV2-F	AAG TAG CGG GAG TGG TAG GA	ORF2
PCV2-R	GGG CTC CAG TGC TGT TAT TC	ORF2
PCV2-Pr	FAM-TCC CGC CAT ACC ATA ACC CAG C-TAMRA	ORF2
PCV3-F	TGA CGG AGA CGT CGG GAA AT	REP
PCV3-R	CGG TTT ACC CAA CCC CAT CA	REP
PCV3-Pr	FAM-GGG CGG GGT TTG CGT GAT TT-TAMRA	REP

Remake: ORF2 = Open Reading Frame 2 of PCV2, REP = Replication-associated protein gene of PCV3.

**Table 3:** Nucleotide sequences of primers and probe used for qPCR detection of *Mycoplasma hyopneumoniae*.

Primer/Probe	Nucleotide Sequence (5′–3′)	Description
Mhp183 F	CCA GAA CCA AAT TCC TTC GCT G	Forward primer
Mhp183 R	ACT GGC TGA ACT TCA TCT GGG CTA	Reverse primer
Mhp183 P	FAM-AGCAGATCTTAGTCAAAGTGCCCGTG-BHQ_1	Probe labeled with FAM/BHQ_1

**Table 4:** Spearman’s Rank Correlation Coefficient and Cohen’s Kappa Coefficient.

Statistical Analysis	Correlation Between Positive and Coughing Symptoms	Correlation Between PCV2 Positive and Non-Productive Cough	Correlation Between *Mh* Positive and Non-Productive Cough
Spearman’s rank correlation coefficient	0.40	0.05	0.80
Cohen’s kappa coefficient (95% CI)	0.36 (CI: 0.10–0.62)	0.037 (CI: –0.20–0.27)	0.79 (CI: 0.56–1.00)

## Data Availability

The original contributions presented in this study are included in the article. Further inquiries can be directed to the corresponding author.

## References

[1] Ferrari S., Silva M., Guarino M., Aerts J.M., Berckmans D. (2008). Cough sound analysis to identify respiratory infection in pigs. Comput. Electron. Agric..

[2] Boonsoongnern A., Jirawattanapong P., Lertwatcharasarakul P., Phatthanakunanan S., Poolperm P., Urairong S., Navasakuljinda W., Urairong K. (2012). The prevalence of Mycoplasma hyopneumoniae in commercial suckling pigs in Thailand. World J. Vaccines.

[3] Jantafong T., Sangtong P., Saenglub W., Mungkundar C., Romlamduan N., Lekchareonsuk C., Lekcharoensuk P. (2015). Genetic diversity of porcine reproductive and respiratory syndrome virus in Thailand and Southeast Asia from 2008 to 2013. Vet. Microbiol..

[4] Pieters M.G., Maes D., Zimmerman J.J., Karriker L.A., Ramirez A., Schwartz K.J., Stevenson G.W. (2019). Mycoplasmosis. Diseases of Swine.

[5] Zimmerman J.J., Dee S.A., Holtkamp D.J., Murtaugh M.P., Stadejek T., Stevenson G.W., Torremorell M., Yang H., Zhang J., Zimmerman J.J. (2019). Porcine reproductive and respiratory syndrome viruses (porcine arteriviruses). Diseases of Swine.

[6] Bálint Á., Molnár T., Kecskeméti S., Kulcsár G., Soós T., Szabó P.M., Kaszab E., Fornyos K., Zádori Z., Bányai K. (2021). Genetic variability of PRRSV vaccine strains used in the national eradication programme, Hungary. Vaccines.

[7] Prieto C., Castro J.M. (2000). Pathogenesis of porcine reproductive and respiratory syndrome virus (PRRSV) in gestating sows. Vet. Res..

[8] Thanapongtharm W., Linard C., Pamaranon N., Kawkalong S., Noimoh T., Chanachai K., Parakgamawongsa T., Gilbert M. (2014). Spatial epidemiology of porcine reproductive and respiratory syndrome in Thailand. BMC Vet. Res..

[9] Osemeke O.H., Cezar G.A., Paiva R.C., Moraes D.C.A., Machado I.F., Magalhães E.S., Silva F.P., Mil-Homens M., Peng J., Jayaraman B. (2023). A cross-sectional assessment of PRRSV nucleic acid detection by RT-qPCR in serum, oral swabs, nasal swabs and ear-vein blood swabs under field conditions. Front. Vet. Sci..

[10] Wasilk A., Callahan J.D., Christopher-Hennings J., Gay T.A., Fang Y., Dammen M., Reos M.E., Torremorell M., Polson D., Mellencamp M. (2004). Detection of U.S., Lelystad, and European-like PRRS Viruses and Relative Quantitation in Boar Semen and Serum Samples by Real-Time PCR. J. Clin. Microbiol..

[11] Sanchez R.P., Garcia M.S., Martinez L.C. (2016). Field evaluation of RT-PCR for PRRSV in blood and oral fluids. Swine Health Prod..

[12] Saade G., Deblanc C., Bougon J., Marois-Créhan C., Fablet C., Auray G., Belloc C., Leblanc-Maridor M., Gagnon C.A., Zhu J. (2020). Coinfections and their molecular consequences in the porcine respiratory tract. Vet. Res..

[13] Gillespie J., Opriessnig T., Meng X.J., Pelzer K., Buechner-Maxwell V. (2009). Porcine circovirus type 2 and porcine circovirus-associated disease. J. Vet. Intern. Med..

[14] Segalés J., Allan G.M., Domingo M., Zimmerman J.J., Karriker L.A., Ramirez A., Schwartz K.J., Stevenson G.W. (2019). Circoviruses. Diseases of Swine.

[15] Rottem S. (2003). Interaction of mycoplasmas with host cells. Physiol. Rev..

[16] Chung Y., Oh S., Lee J., Park D., Chang H.H., Kim S. (2013). Automatic detection and recognition of pig wasting diseases using sound data in audio surveillance systems. Sensors.

[17] Shen W., Ji N., Yin Y., Dai B., Tu D., Sun B., Hou H., Kou S., Zhao Y. (2022). Fusion of acoustic and deep features for pig cough sound recognition. Comput. Electron. Agric..

[18] Yamsakul P., Yano T., Na Lampang K., Khamkong M., Srikitjakarn L. (2022). Infrared temperature sensor for use among sow herds. Vet. Integr. Sci..

[19] Tzanidakis C., Simitzis P., Arvanitis K., Panagakis P. (2021). An overview of the current trends in precision pig farming technologies. Livest. Sci..

[20] Yamsakul P., Yano T., Na Lampang K., Khamkong M., Srikitjakarn L. (2023). Classification and correlation of coughing sounds and disease status in fattening pigs. Vet. Integr. Sci..

[21] Kleiboeker S.B., Schommer S.K., Lee S.-M., Watkins S., Chittick W., Polson D. (2005). Simultaneous detection of North American and European porcine reproductive and respiratory syndrome virus using real-time quantitative reverse transcriptase–PCR. J. Vet. Diagn. Investig..

[22] Franzo G., Segalés J. (2018). Porcine circovirus 2 (PCV-2) genotype update and proposal of a new genotyping methodology. PLoS ONE.

[23] Yuan W., Li J., Li L., Sun M., Zheng Y., Qi Y., Sun J., Song Q. (2014). Rapid detection of porcine circovirus type 2 by TaqMan-based real-time polymerase chain reaction assays. Int. J. Appl. Res. Vet. Med..

[24] Silva P.A.P.S., Storino G.Y., Ferreyra F.S.M., Zhang M., Fano E., Polson D., Wang C., Derscheid R.J., Zimmerman J.J., Clavijo M.J. (2022). Cough associated with the detection of Mycoplasma hyopneumoniae DNA in clinical and environmental specimens under controlled conditions. Porc. Health Manag..

[25] Berckmans D., Moshou D., Chen L., Ramon H. (2008). A sound-based monitoring system for the detection of coughing in pigs. Comput. Electron. Agric..

[26] Zhao J., Li X., Liu W.H., Gao Y., Lei M.G., Tan H.Q., Yang D. (2020). DNN–HMM based acoustic model for continuous pig cough sound recognition. Int. J. Agric. Biol. Eng..

[27] Goldwater S., Jurafsky D., Manning C.D. (2009). Which words are hard to recognize? Prosodic, lexical, and disfluency factors that increase speech recognition error rates. Speech Commun..

[28] Wathes C.M., Kristensen H.H., Aerts J.-M., Berckmans D. (2021). A systematic review on validated precision livestock farming technologies for pig production and its potential to assess animal welfare. Front. Vet. Sci..

[29] Segalés J., Domingo M. (2002). Porcine circovirus 2 lung disease: Characterized by respiratory distress and dyspnea. Int. Anim. Health J..

[30] Yang S., Oh T., Park K.H., Cho H., Chae C. (2022). A dual swine challenge with Porcine Circovirus Type 2 (PCV2) and Mycoplasma hyopneumoniae used to compare vaccine types. Front. Vet. Sci..

[31] Opriessnig T., Meng X.J., Halbur P.G. (2004). Experimental reproduction of PMWS in pigs with dual infection of Mycoplasma hyopneumoniae and PCV2. Vet. Pathol..

[32] Maes D., Segalés J., Meyns T., Sibila M., Pieters M., Haesebrouck F. (2008). Control of Mycoplasma hyopneumoniae infections in pigs. Vet. Microbiol..

[33] Sibila M., Pieters M., Molitor T., Maes D., Haesebrouck F., Segalés J. (2009). Current perspectives on the diagnosis and epidemiology of Mycoplasma hyopneumoniae infection. Vet. J..

[34] Babbs C.F. (2020). Biomechanical Models of Cough Sounds in Pneumonia: Mechanisms Underlying Sound-Based Diagnosis in Low-Resource Settings. Purdue e-Pubs. https://docs.lib.purdue.edu/cgi/viewcontent.cgi?article=1022&context=bmewp.

[35] Chung Y., Lee H., Kang H., Park S.J., Choi Y., Cho K.H. (2021). Sound-based detection of swine respiratory disease using deep learning. Comput. Electron. Agric..

[36] Saito M., Takemoto M., Tanaka S., Oishi K. (2023). Validation of a real-time monitoring system for pig cough detection in commercial farms using AI and its application to early disease surveillance. Animals.

